# Scalable activated carbon/graphene based supercapacitors with improved capacitance retention at high current densities

**DOI:** 10.3906/kim-2012-39

**Published:** 2021-06-30

**Authors:** İ. Işıl GÜRTEN İNAL

**Affiliations:** 1 Department of Chemical Engineering, Faculty of Engineering, Ankara University, Ankara Turkey

**Keywords:** Activated carbon, electrochemical exfoliated graphene, supercapacitor, capacitance stability, pouch cell

## Abstract

Scalable, highly stable supercapacitor electrodes were developed from the mixture of a tea factory waste based activated carbon (AC) and a low-cost electrochemical exfoliated graphene (EEG). The hybrid electrodes showed notably enhanced stability at high current densities. The AC sample was prepared by chemical method and exposed to a further heat treatment to enhance electrochemical performance. Graphene used in the preparation of hybrid electrodes was obtained by direct electrochemical exfoliation of graphite in an aqueous solution. Detailed structural characterization of AC, EEG, and hybrid material was performed. The original electrochemical performances of AC and EEG were examined in button size cells using an aqueous electrolyte. The hybrid materials were prepared by mixing AC and EEG at different mass percentage ratios, and tested as supercapacitor electrodes under the same conditions. Capacitance stability of the electrodes developed from AC:EEG (70:30) at high currents increased by about 45% compared to the original AC. The highest gravimetric capacitance (110 F/g) was achieved by this hybrid electrode. The hybrid electrode was scaled up to the pouch size and tested using an organic electrolyte. The organic electrolyte was preferred for scaling up due to its wider voltage ranges. The pouch cell had a gravimetric capacitance of 85 F/g and exhibited as good performance as the coin cell in the organic electrolyte.

## 1. Introduction

Research on high energy and power energy storage systems has gained significant momentum over the past decade. Supercapacitors are the electrochemical energy storage systems designed to store and transfer energy quickly. While supercapacitors can store hundreds of times more energy than conventional capacitors, they can transfer this energy approximately 10 times faster than electrochemical batteries. Thanks to the appropriate cell design, it is possible to obtain both high energy and power. Supercapacitors can be used in emergency energy applications, portable electronic devices, industrial power requirements, and some hybrid applications [1–3]. The performance of supercapacitors mainly depends on the properties of electrode materials and the electrode-electrolyte interface [4]. Porous carbon materials are more advantageous than other electrode materials (conductive polymers and metal oxides) due to their low-cost, established manufacturing techniques, tunable surface area, pore size distribution, pore shape, and surface chemistry [5,6]. Since the charge storage mechanisms of porous carbon materials are based on the principle of reversible electrical double layer formation, their capacitance stability, cycle life, and rate capacity are higher than pseudo-capacitive materials (conductive polymers and metal oxides).

Activated carbons are the only supercapacitor electrode material that succeeded in commercialization due to its high surface area, controllable pore size distribution, and low-cost compared to other carbon materials [7–9]. A wide range of raw materials is used for the production of activated carbon. These can be classified as the lignocellulosic (wood, agricultural wastes, fruit seeds), fossil (coal, lignite, peat, petroleum coke), and polymeric materials [10–15]. Activated carbon production is made by physical and chemical activation methods. The chemical activation method is the most preferred one since it leads to develop higher pore volume and surface area [3]. There are certain activation chemicals used for this purpose such as KOH, H_3_PO_4_, K_2_CO_3_, and NaOH [10,13,15]. The type of the starting material and activation agent directly affect the surface properties of the final product. Among all starting materials, waste biomasses are the most popular ones due to their low-cost and sustainability. In the literature, there are various waste biomasses such as tea waste [16], rice husk [17], waste coffee beans [18], and cumin plant [19] which used as precursors for the activated carbons as supercapacitor electrode materials. Some studies on the use of biomass-based activated carbons as supercapacitor electrodes are summarized in Table 1. The specific capacitances of different biomass-derived carbon electrodes vary greatly depending on the type of biomass, production method, and the conditions of the electrochemical characterization.

**Table 1:** Some biomass-based activated carbons tested as supercapacitor electrode.

Precursor	Activator	SBET (m2/g)	Capacitance(F/g)	Current density/scan rate/Freq.	Electrolyte	Ref.
Rice husk	NaOH	1886	210	0.2 mA/g	3 M KCl	[44]
Fir wood	H2O	1131	140	25 mV/s	0.5 M H2SO4	[45]
Pistachio shells	KOH	1096	120	10 mV/s	0.5 M H2SO4	[46]
Rotten carrot	ZnCl2	1155	135	10 mHz	6 M KOH	[47]
Bamboo	KOH	1251	260	1 mA/cm2	30% H2SO4	[48]
Banana fibers	ZnCl2	1097	74	500 mA/g	1 M Na2SO4	[49]
Corn grains	KOH	3199	257	1 mA/cm2	6 M KOH	[50]
Corn syrup	H2O	1473	168	0.2 A/g	6 M KOH	[51]
Sugar cane bagasse	ZnCl2	1788	300	250 mA/g	1 M H2SO4	[52]
Cassava peel waste	KOH	1352	153	-	0.5 M H2SO4	[53]
Argan seed shell	KOH/melamin	2062	355	125 mA/g	1 M H2SO4	[14]
Tea factory waste	H3PO4K2CO3	13271125	123203	1 mA/cm2	1 M H2SO4	[16]
Demineralized cumin plant	H3PO4	1472	155	1.5 mA/cm2	1 M H2SO4	[19]
Tea factory waste	ZnCl2	923	140100	0.1 A/g	6 M KOH1 M Et4NBF4	[54]
Pine cone	ZnCl2	2007	87	10 mV/s	1 M Et4NBF4	[55]
Tea factory waste	H3PO4	804	11088	0.1 A/g	6 M KOH1 M Et4NBF4	[This work]

In the use of activated carbon in energy storage applications, surface area, pore structure (size, volume, and shape), and resistance/conductivity behavior of the porous carbon material are the most important properties that affect electrochemical performance [3]. As the surface area increases, the capacitance increases due the higher amount of charge accumulated on the electrode surface. However, high surface area alone is not sufficient for high capacitance. The pore structure of the carbon electrode has also great importance. It is also crucial that the pores are to be ‘open’ as well as their sizes. Not all pores on the surface are electrochemically accessible. If the electrolyte cannot enter the closed pores and the electrode-electrolyte contact is not maintained sufficiently, the electrical double layer formation is prevented. In capacitor applications, the micro/mesopore ratio of the porous electrode is also essential. Many studies in the literature on the effect of pore size on electrode performance exist [16,20–22]. However, the effect of pore size on the electrochemical performance has not yet been elucidated due to the complex structure of the porous materials used as electrodes and the electrode-electrolyte interface. The type and amount of surface functional groups in carbon electrodes also changes the electrochemical and double layer properties significantly [23,24]. These properties can be controlled by changing the conditions of the carbonization and activation steps [2]. Despite all advantages of using activated carbons as electrode materials, low capacitance stability at high current densities is the main problem of activated carbon based supercapacitors and limits their commercial applications [9]. 

On the other hand, graphene-based supercapacitors have recently attracted considerable attention due to the superior electrical properties of the material (high electrical conductivity), high theoretical surface area (which is related to the number of graphene layers, not porosity), and thermal/chemical stability [25]. Graphene has been prepared by traditional high-cost, high-temperature or high-polluting methods such as chemical vapor deposition (CVD) and chemical exfoliation of graphite [26–31]. However, the electrochemical exfoliation of graphite is quite simple, cheap, and clean graphene production technique compared to the others which make the product a suitable material for high-power supercapacitor applications [32,33]. Thanks to its unique properties, graphene is considered as a material that will solve the existing problems and provide the expected improvement in supercapacitor research. However, its high production cost and low electrochemically accessible surface area due to the tendency of restacking of graphene layers limits its use as supercapacitor electrode material [34]. Therefore, developing hybrid activated carbon/graphene electrode materials can be a solution to overcome the drawbacks mentioned above. There are many studies in which graphene have been used as an electrode material along with other carbon materials such as carbon activated carbon-graphene [27,32,35–37] nanotube-graphene [38,39], carbon spheres-graphene [40], and carbon black-graphene [41]. The graphene used in these studies was generally synthesized on carbon material by high cost and high-polluting chemical methods or simultaneous reduction during carbon material production. 

In addition to all this recent improvement in carbon based supercapacitor research, there is an increasing interest for the fabrication of structured carbon based electrodes with different sizes from micro scale to large scale due to their immense potential in diverse commercial applications such as miniaturized, portable electronic devices, electric vehicles, and large scale industrial equipment [32,42,43].

The main objective of this study is to develop the waste tea based activated carbon/electrochemically exfoliated graphene scalable hybrid supercapacitor electrodes showing better stability at high currents in comparison to the AC based electrodes. To the best of our knowledge, a waste biomass based AC/EEG scalable supercapacitor electrode showing an improved stability has not been reported before. 

## 2. Materials and methods

### 2.1. Preparation of the starting material for the activated carbon production

Tea factory waste obtained from Ulusoy Tea Factory (Rize) in Turkey was used as the starting material for the production of activated carbon. It was crushed by a high speed (20,000 rpm) laboratory mill (IKA M20 Universal Mill). The crushed particles were sieved under 500 µm particle sizes, and then used for the production of the activated carbon sample. 

### 2.2. Production of the activated carbon 

Activated carbon sample was produced by the chemical activation method using H_3_PO_4_ as the chemical activation agent. It was previously showed that the microwave pre-treatment in the production of activated carbon from waste tea using H_3_PO_4_ significantly increased the surface area and pore volume of the final product [13]. The waste tea was impregnated with H_3_PO_4_ and then directly subjected to the microwave (MW) pre-treatment for 30 s in a Teflon container by using a domestic microwave oven (Vestel, MDG-620). The input power of the microwave equipment was set at 900 W and the microwave frequency used was 2.45 GHz). Heat treatment of the microwave pre-treated sample was applied in a quartz reactor under N_2_ atmosphere using a temperature programmable furnace (Nabertherm RSR 120/500/11). The production parameters were directly selected from the previous study by the author [16]. The production conditions of the activated carbon were summarized in Table 2. The resultant solid sample was washed with hot distilled water until the pH neutral, and then dried (110 ºC for 24 h).

**Table 2:** Experimental conditions for the activated carbon production.

H3PO4/Waste tea	3.0 (weight/weight)
MW pre-treatment power and duration	900 W, 30 s
Carbonization/activation duration	60 min
N2 flow rate	0.15 L/min
Heating rate	10 °C/min
Carbonization/activation temperature	450 °C

### 2.3. Further heat treatment to the activated carbon sample

It was showed that a further heat treatment applied to the activated carbon causes a significant change in surface properties and increases the conductivity of the sample leading to a significant enhancement in the electrochemical performance by the author’s previous study [54]. In the present study, the further heat treatment was directly applied to the activated carbon sample in an inert atmosphere for 1 h at 800 °C. The resultant carbon sample was labelled as AC, and used as the supercapacitor electrode material alone and along with the graphene produced.

### 2.4. Preparation of the electrochemical exfoliated graphene (EEG)

The low-cost graphene used as electrode material along with AC was prepared by anodic electrochemical exfoliation of graphite foil in a aqueous solution of 0.1 M (NH_4_)_2_SO_4_ [33]. The exfoliation was carried out in a 100 mL glass electrochemical test cell filled with the electrolyte solution. The graphite foil (anode) was cut into the 1 cm x 4 cm size and used as the working electrode. The counter electrode was platinum (cathode). During the exfoliation process, a constant voltage of +10 V was applied to the working electrode. The graphene particles started to exfoliate and passed into the solution medium, and the process continued until the graphite foil was entirely consumed (2 h). The exfoliated product was washed with distilled water until pH neutral by vacuum filtration using a PTFE membrane. In order to separate un-exfoliated graphite from the graphene, the mixture was centrifuged in distilled water at 1500 rpm for 10 min and then kept for 48 h, allowing the un-exfoliated graphite to precipitate. The graphene was collected from the suspended part by vacuum filtration and dried at 105 °C for 24 h. The resulting solid film was dispersed in ethanol by ultrasonication and then dried. Finally, the sample was milled to obtain a powder form. The production process was repeated to get a sufficient amount of graphene for the electrode preparation. The product yield was about 70% for each process. 

The hybrid electrode materials were prepared by simply mixing the AC and EEG powders in different ratios. The details of the preparation of the hybrid electrode materials were given in Section 2.6. 

### 2.5. Product characterization

Since the characterization of the tea factory waste was given in detail in the previous studies [13,15], only the structural characterization of AC, EEG, and AC: EEG hybrid electrode materials are presented here.

**Surface area and pore size distribution analysis:** The BET surface area and pore size distribution of AC, EEG and the hybrid electrode materials were determined from N_2_ (99.999% purity) adsorption data by using a Quantachrome Nova 2200 series surface area and a pore size analyzer. BET surface area values were calculated by multi-point method [56]. Pore volumes and pore size distributions were calculated according to the “non local density functional theory” (NLDFT) method by using N_2_ adsorption data at 77 K. It was assumed that the sample consists of both cylindrical and long-narrow slit shaped pores (slit/cylinder). Total pore volume (micro + meso + macro if available) was determined at the relative pressure (P/Po) of 0.99. The mesopore volume (2–50 nm) was calculated by subtracting the micropore volume (at 2 nm) from the total pore volume by interpolating the total pore size distribution data. 

**XPS (X-ray photoelectron spectroscopy) analyses:** The elemental compositions of AC, and EEG were determined by using a Kratos Axis Ultra DLD x-ray photoelectron spectrometer with a monochromatic Al Kα X-ray source. The distributions of oxygen functionality were examined by deconvolution of O 1s peak using an iterative least squares algorithm (Casa XPS software) with a Gaussian–Lorentzian peak shape. The percentages of the functional groups are calculated by the software based on the areas of the deconvoluted peaks.

**Raman analyses:** Structural characterization of AC and EEG was performed with a Renishaw in a Via Raman microscope using a 532 nm excitation laser operated at 10 mW power.

**SEM (scanning electron microscope) analyses:** The surface morphology of AC, EEG, and the best performing hybrid electrode material was examined with FEI QUANTA 450 Field Emission SEM. 

### 2.6. Preparation of the supercapacitor electrodes

The supercapacitor performance of AC and AC/EEG hybrid electrode materials was tested in coin cell size. The best performing hybrid electrodes were scaled up to the pouch cell. The preparation of the coin and pouch type cells is summarized below.

AC based supercapacitor electrode mixture consisted of 85% AC, 10% carbon black, and 5% polyvinylidene difluoride in weight as the active material, conductive additive, and polymeric binder, respectively. On the other hand, since the use of a second conductive material (carbon black) would be misleading in terms of the contribution of EEG to the electrode performance, carbon black was not used in hybrid electrodes. Therefore, AC/EEG based hybrid electrodes consisted of 95% AC/EEG mixture as the active material and 5% polyvinylidene difluoride as the binder. 

N-methyl-2-pyrrolidone (NMP) was used as the solvent of the binder. The hybrid active materials were prepared by the three different mass ratios (AC: EEG) of 90:10, 70:30, and 50:50. The required amount of Triton X-100 surfactant was added to the electrode mixture to prevent the graphene particles from agglomerating and increase the electrode wettability. Agglomeration of powdered carbon materials is a main problem in electrochemical applications to cause the insufficient wettability of the electrode by the electrolyte. Although this problem is addressed in the current literature on graphene based supercapacitors, it has been almost neglected in the studies on the activated carbon-based supercapacitors. The addition of the surfactant (Triton X-100) to the electrode mixture leads to the solids disperse well in the mixture and hence improve the wettability of the electrodes. The electrode mixture was sprayed on to a Ni foam current collector, and then dried in a vacuum oven at 85 °C for 24 h to remove NMP. In order to prepare coin cells, dried electrodes were cut into the circular shape (16 mm diameter) using an electrode cutting machine. An electrolyte soaked paper separator (Nippon Kodoshi) is placed between two identical electrodes to prevent the direct contact between them. Finally, the electrodes were put into the stainless steel cases and then sealed by using a crimping machine. 

**Figure 1:**
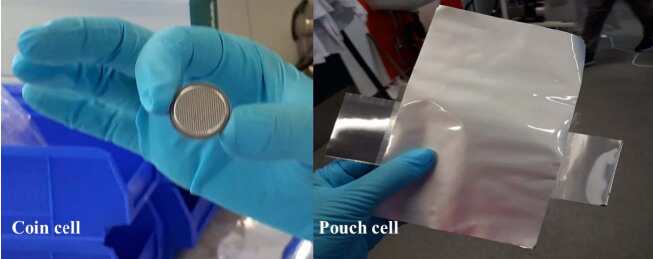
Coin and pouch size cells.

The hybrid electrodes that showed the best electrochemical performance were scaled up to the pouch size. For the pouch cell preparation, the coated Ni foam were cut into the size of 5 cm^2^. The cell were stacked to contain two positive and two negative electrodes and sealed with a special polymer laminated aluminum foils using a laboratory-scale pouch cell crimping machine. A coin and pouch cells are shown in Figure 1.

In order to test the coin cells, both aqueous (6 M KOH) and organic electrolytes (1 M Et4NBF4/acetonitrile) were used. Since the operating voltage ranges of the organic electrolytes are much larger than the aqueous ones, they are more preferable for the commercial applications. All organic cells were assembled in an argon-filled glove box.

### 2.7. Electrochemical tests of the supercapacitor cells

Cyclic voltammetry (CV), galvanostatic charge/discharge (GCD), and impedance spectroscopy (EIS) techniques were used for the electrochemical characterization of the supercapacitor cells. All these analyses were carried out by using a Gamry Reference 3000 potentiostat/galvanostat. CV analysis was performed at different scan rates of 10 **–** 200 mV/s. The charge storage capacities of the cells were examined by GCD analysis at different constant current densities of 0.1, 0.2, 0.5, 1.0, 2.0, 5.0, 7.0, 10 A/g (up to 20 A/g for the pouch cell). The long term stability of the pouch cell was tested over 10,000 cycles at a constant current density of 1 A/g. Impedance analysis, which gives information about the resistance behaviour of the cell, was carried out in the frequency range of 0.01 **–** Hz100 kHz. Voltage windows for the 6 M KOH and 1 M Et_4_NBF_4_/acetonitrile electrolytes were 0.0 **–** 0.8 V and 0.0 **–** 2.0 V, respectively.

The gravimetric capacitances of the electrodes were calculated according to the Eq. (1) using the GCD analysis data.

C_spec_=2I∆t/m∆V (1) 

Here; C_spec_ (F/g) is the gravimetric capacitance, I (A) is the current, Δt (s) is the discharge time, ΔV (V) is the potential window during the discharge process, and m (g) is the mass of a single electrode. 

Coulombic efficiencies of the pouch cell calculated using the Eq. (2)

η=(t_d_/t_c_ )*100 (2)

Here; t_d_ and t_c_ are the duration of the charge and discharge processes, respectively.

## 3. Results and discussion

### 3.1. Material characterization

#### 3.1.1. Surface area and pore analyses 

**Figure 2:**
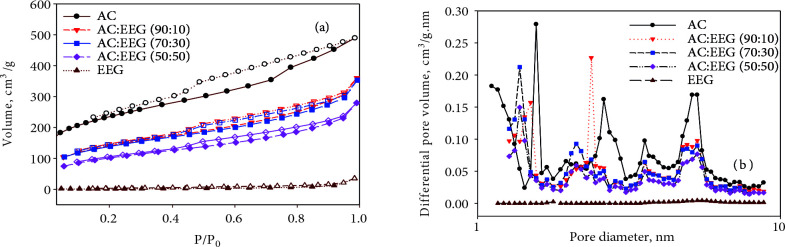
(a) N2 adsorption-desorption isotherms and (b) pore size distribution curves (NLDFT) of AC, EEG, and the hybrid electrode materials.

N_2_ adsorption-desorption isotherms and pore size distributions of AC, EEG, and all three hybrid electrode materials (AC: EEG (90:10), (70:30), and (50:50) containing polymeric binder are given in Figure 2 (a) and (b), respectively. According to the IUPAC, International Pore Size Classification, the adsorption-desorption isotherm of a porous solid is directly related to the pore size distribution and pore structure of the material. The isotherm of AC exhibits typical Type IV hysteresis behavior (mesoporous solid). In general, many activated carbons exhibit Type IV hysteresis that has a composite nature. The initial part of the reversible micropore filling is followed by multilayer physisorption and capillary condensation in this type of isotherm [57]. On the other hand, the isotherm of EEG represents a nonporous solid behavior. As expected, the isotherms of the hybrid electrode materials containing AC, EEG and the polymeric binder (PVdF) show that they have a lower surface area than AC. The presence of the binder in the hybrid electrode materials reduced the N_2_ adsorption on the surface to some extent. The BET surface area, pore volumes and the fractions of all the samples are given in Table 3. The findings in Table 3 confirm the behavior of the isotherms and pore size distribution curves of the samples.

**Table 3:** BET surface area, pore volumes and fractions of AC, EEG, and hybrid electrode materials containing binder

	SBET(m2/g)	Vtotala (cm3/g)	Vmicrob (cm3/g)	Vmesoc (cm3/g)	Vmicro(%)	Vmeso(%)
AC	803.94	0.716	0.177	0.539	24.72	75.28
AC:EEG (90:10)	491.60	0.377	0.041	0.336	10.87	89.13
AC:EEG (70:30)	481.71	0.353	0.060	0.293	16.99	83.01
AC:EEG (50:50)	357.00	0.278	0.038	0.240	13.67	86.33
EEG	6.15		-	-	-	-

#### 3.1.2. XPS analyses 

**Figure 3:**
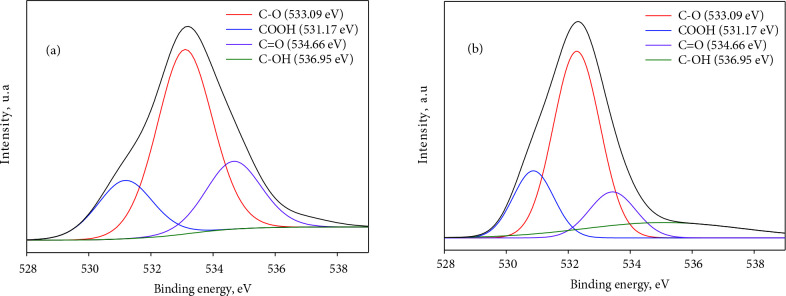
Fitted results of XPS O 1s spectra of (a) AC and (b) EEG.

The elemental content and the concentration of the oxygenated functional groups of AC and EEG are given in Table 4. The O 1s spectra of AC and EEG are also presented in Figure 3. The presence of N and S content in EEG is due to the electrolyte used in the electrochemical exfoliation process. The oxygen content of AC is relatively lower than EEG probably because of the further heat treatment applied to the carbon sample. The previous reports were considered to interpret the binding energy values of the surface groups of the samples [16,58]. The O 1s spectra display peaks of oxygen in carbonyl (C=O) and carboxylic groups (O=C-OH), oxygen in hydroxyl or ethers (C-O), and chemisorbed oxygen in H_2_O and/or C-OH. The higher oxygen content of EEG (especially chemisorbed oxygen in H_2_O and/or C-OH groups) is mainly due to the water molecules remaining between the graphene layers during graphene production by electrochemical exfoliation and the oxidation of graphite during exfoliation [33]. The oxygen functional groups, especially carboxyl and carbonyl groups enhance the electrochemical performance of the electrode material by improving wettability of the electrode and the electrolyte diffusion rate into the electrode. Moreover, they may increase the specific capacitance by an additional pseudocapacitance effect in a 6 M KOH aqueous electrolyte [59]. As seen in Table 4, while the concentration of the carboxyl groups on the EEG surface is higher than AC, the concentration of the carbonyl groups on the AC surface is higher than EEG. The synergetic interaction of these groups may be one of the possible reasons for the enhanced electrochemical performance obtained by hybrid electrodes.

**Table 4:** Elemental composition and oxygenated groups concentration of AC and EEG.

	AC		
Element	Concentration, %	Surface group	Concentration, %
C 1s	90.75	C=O	21.60
N 1s	1.12	COOH	18.34
O 1s	7.35	C-OH	2.55
P 2p	0.78	C-O	57.51
	EEG		
Element	Concentration, %	Surface group	Concentration, %
C 1s	88.20	C=O	16.60
N 1s	1.00	COOH	23.80
O 1s	10.60	C-OH	11.90
S 2p	0.20	C-O	47.70

#### 3.1.3. SEM analyses 

**Figure 4:**
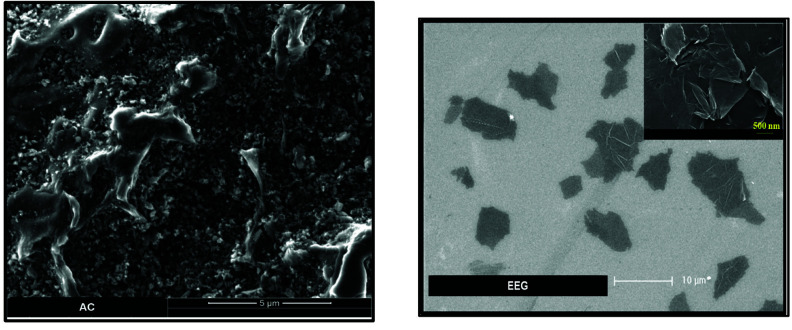
SEM images of AC and EEG.

SEM image of AC and EEG are given in Figure 4. Micro and mesopores on the surface of AC are too small to observe by SEM technique. However, the presence of cracks and holes in various sizes and the roughness of the surface provide information about the porosity and surface morphology. It can be seen that AC has a fairly rough and heterogeneous surface morphology which may a result of a further heat treatment applied to the carbon sample. On the other hand, the presence and the size of the graphene flakes were proven by the SEM image of EEG. Moreover, the layered and flat surface of the graphene flakes without any significant roughness can be clearly observed in the inset image.

#### 3.1.4. Raman analyses

**Figure 5:**
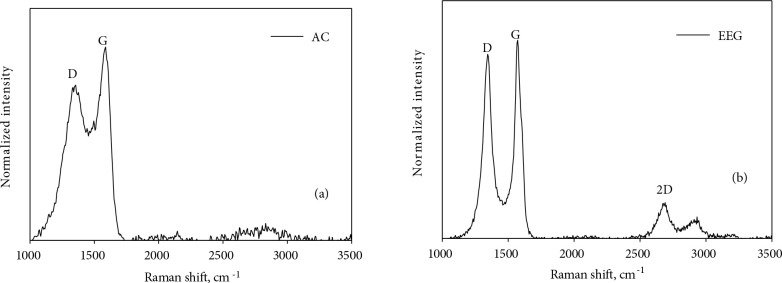
Raman spectra of (a) AC and (b) EEG.

The Raman spectra of AC and EEG are presented in Figure 5. The spectrum of AC has two main peaks at around 1300 cm^−1^ and 1530 cm^−1^, which are assigned to the well-known D and G bands, respectively. While, the D band expresses the defects caused by the sp^3^ hybridized carbon atoms, the G band represents the C-C bond vibration in the graphene layers in the sp^2^ electronic structure. The ratio of intensity between the D and G band (I_D_ /I_G_) gives the information about the graphitic degree and the intensity of the surface functional groups of the activated carbon. The (I_D_/I_G_) ratio of AC is found to be 0.80. 

The Raman spectrum of EEG presents characteristic peaks of D, G, and 2D bands at around 1350 cm^−1^, 1580 cm^−1^, and 2680 cm^−1^, respectively. The observation of the 2D peak is an indication of structural transformation from graphite to graphene heterostructure after electrochemical exfoliation [60]. The (I_D_/I_G_) peak ratio of EEG is 0.91. The prominent D peak is an indicator of a structural defect and functionality on the surface coming from oxygenation of graphitic carbons during the electrochemical exfoliation process [61]. The Raman spectrum of the synthesized EEG showed the structural similarity to the some other electrochemically exfoliated graphene materials in the literature [32,62,63]. Moreover, the number of layers of graphene can be estimated by the shape and intensity of the 2D peak. The Raman spectrum of EEG shows the typical behaviour of a 5 layer (or above) graphene which is calculated from the value of (I_2D_/I_G_) [60]. Finally, the lower (I_D_/I_G_) peak ratio of AC than EEG is possibly due to a decrease in surface functionality of AC as a result of the further heat treatment procedure applied proven by the XPS analyses results previously. 

### 3.2. Electrochemical characterization of AC and AC/EEG coin size electrodes

**Figure 6:**
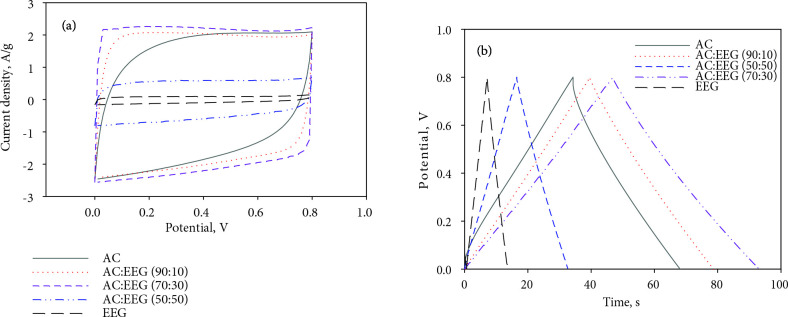
(a) CV curves at 50 mV/s scan rate, and (b) GCD curves at a constant current density of 1 A/g of AC, EEG, and AC/EEG electrodes in 6 M KOH.

**Figure 7:**
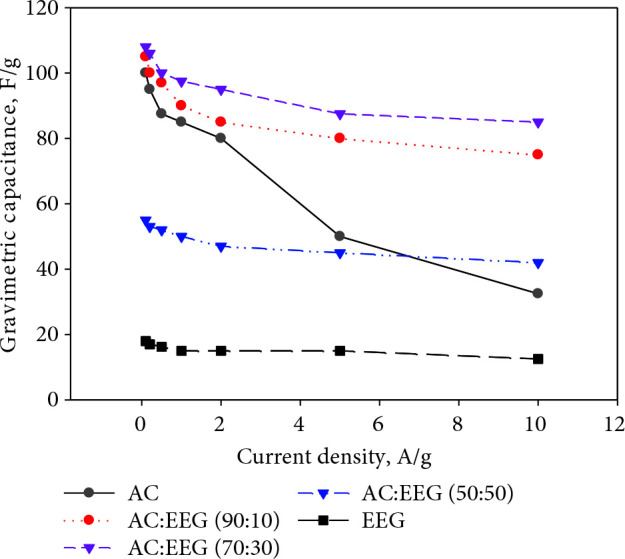
Gravimetric capacitances of AC, EEG, and AC/EEG electrodes at different constant current densities in 6 M KOH.

Hybrid supercapacitor electrodes were prepared by mixing AC and EEG in three different mass percentage ratios of 90:10, 70:30, and 50:50 (AC:EEG) and tested as the coin size cells in aqueous media (6 M KOH). The CV the GCD curves of the hybrid electrodes along with AC and EEG are given in Figures 6 (a) and (b), respectively. The CV curves of the electrodes consisting of 10% and 30% of EEG represent excellent rectangular shape proving that the increased reversibility and rate capability of the cell. When the mass ratio of EEG in the electrode reached 50%, the gravimetric capacitance of the electrodes decreased dramatically. This is probably due to the decrease in the surface area of the electrode. Figure 7 shows the gravimetric capacitances of all the electrodes at different constant current densities. The maximum gravimetric capacitance values of the electrodes containing %10, %30, and %50 of EEG were 100 F/g, 110 F/g, and 55 F/g, respectively. On the other hand, the maximum gravimetric capacitances of the electrodes containing only AC and EEG were 100 F/g and 18 F/g, respectively. The low capacitance value of graphene based electrodes is due to its nonporous structure (low surface area). 

Remarkably, the electrodes prepared from AC significantly lost their initial capacitance with the increasing current density (65% loss). The poor capacitance stability at high currents is a major disadvantage of activated carbon based supercapacitor electrodes which restricted their usage in commercial applications [9,32]. A significant loss of capacitance at high currents has also been reported before in many studies on biomass-derived carbon supercapacitor electrodes [47,51,54].

**Figure 8:**
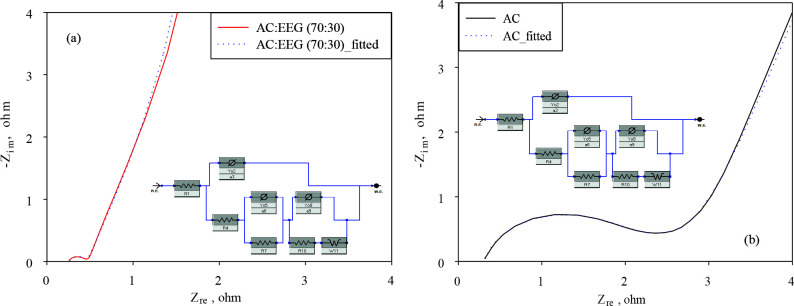
Impedance curves of AC and AC/EEG (70:30) electrodes in 6 M KOH.

A 10% increase in gravimetric capacitance was achieved by the hybrid electrodes containing 30% EEG (70:30). Moreover, as targeted, the capacitance loss at high currents significantly reduced with this hybrid electrodes (only 18%). These findings were supported by some previous studies on graphene enhanced activated carbon based supercapacitor electrodes [35,36]. The impedance curves of AC and the best performing hybrid electrodes (70:30) are presented in Figure 8 as the Nyquist plots. A significant decrease in the internal resistance of the hybrid electrode material was achieved. The primary reason for this decrease is that graphene is to contribute to the faster and more efficient formation of electrical double layer by creating paths that will allow the movement of charges between the activated carbon particles and the current collector thanks to its high conductivity [32,36]. 

### 3.3. Electrochemical characterization of the pouch size electrodes

**Figure 9:**
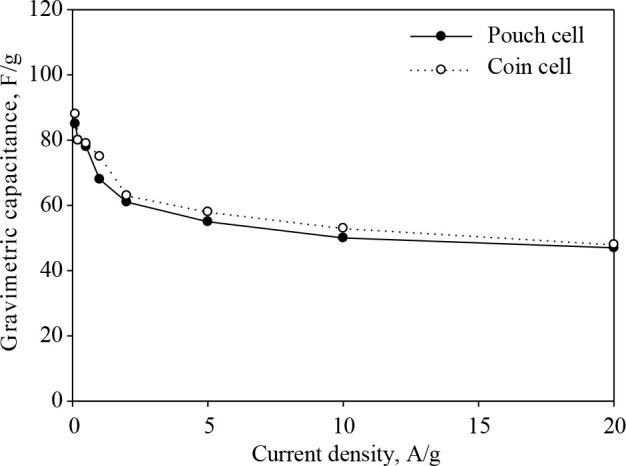
Gravimetric capacitances of coin and pouch size electrodes in 1 M Et4NBF4.

**Figure 10:**
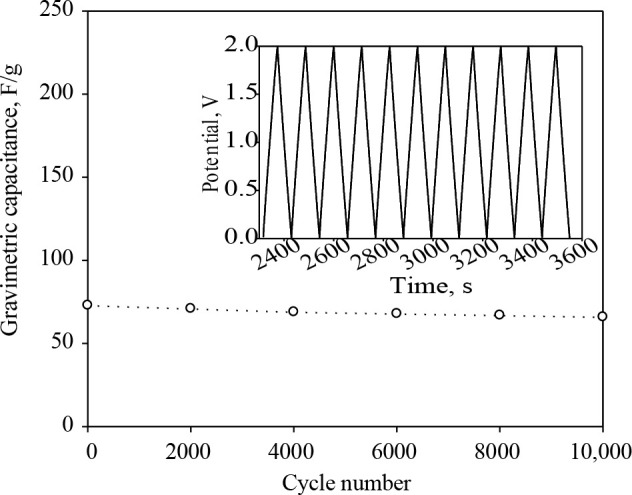
The gravimetric capacitance of the pouch size electrodes over 10,000 cycles in 1 M Et4NBF

The best performing hybrid electrodes (70:30) was scaled up to the pouch size. Since the polymer laminated aluminum foil, which was used for the fabrication of the pouch cell, may give undesired side reactions in the aqueous electrolyte, it was tested in the organic electrolyte (1 M Et_4_NBF_4_/acetonitrile). Furthermore, as mentioned in Section 2.6, organic electrolytes are more preferable in commercial applications thanks to their wide potential ranges. In order to show the effect of the scaling up on the electrochemical performance, a coin size cell was prepared from the best performing hybrid material and tested in organic electrolyte as well. Figure 9 shows the gravimetric capacitances of coin and pouch size electrodes developed from hybrid material at different current densities. The maximum gravimetric capacitance values of the organic coin and pouch size hybrid electrodes were 88 F/g and 85 F/g, respectively. Since the resistance of organic electrolytes is higher than aqueous ones, it was an expected result to obtain lower capacitance values compared to the aqueous one [58]. Both coin and pouch size hybrid electrodes exhibited notably stable behavior at the high current densities. The capacitance of the pouch size electrodes was slightly lower than coin size electrodes due to the decrease in the ion accessible area with increasing electrode size [32,54]. The cyclic stability of the pouch cell over 10,000 charges/discharge cycles at a constant current density of 1 A/g is given in Figure 10. The pouch cell developed from the hybrid material maintained its capacitance over 90% after 10,000 cycles of charging and discharging. The symmetrical charge/discharge curves also confirmed that the hybrid electrode material exhibited highly reversible, ideal electrical double layer behavior.

The gravimetric capacitance values varying cycle number at the current density of 1 A/g and the coulombic efficiencies of the pouch size hybrid electrodes tabulated in Table 5.

**Table 5:** The gravimetric capacitance values varying cycle number at the current density of 1 A/g and the coulombic efficiencies of the pouch size hybrid electrodes.

Cycle number	Cspec (F/g)	Coulombic eff. (%)
1	75	97
1000	73	96
5000	70	94
10,000	68	93

### 3.4. Morphological characterization of the hybrid electrode material

**Figure 11:**
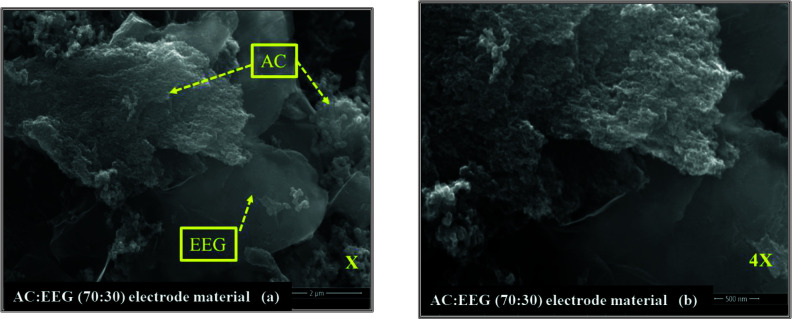
SEM images of the best performing hybrid material taken from the same spot at (a) the magnification of x and (b) 4x.

The SEM images of the best performing hybrid electrode material taken from the same spot at different magnifications are presented in Figures 11 (a) and (b). AC and EEG are clearly distinguished from one another. It is noteworthy that conductive EEG layers form connections (paths) between AC particles. The charges may have moved faster thanks to the highly conductive path between the particles and the metal surface, leading an increase in the stability and the rate capacity of the electrodes.

## 4. Conclusions

Low stability at high currents is one of the most important drawbacks of the activated carbon-based supercapacitor. In this study, an exceptional improvement in capacitance stability at high currents was achieved by a low-cost hybrid material prepared with different mass ratios by using a waste biomass-based activated carbon and an electrochemically exfoliated graphene. The best performing hybrid material was successfully scaled up to the pouch size without loss of performance.

 The activated carbon sample (AC) was developed from the tea factory waste by a further heat treatment step following the chemical method. The graphene (EEG) used to prepare hybrid electrode material was synthesized from graphite by using a facile electrochemical exfoliation technique. The detailed structural characterization of AC and EEG were performed.

It has been concluded that AC and EEG particularly have different morphology and surface chemistry. The nonporous EEG flakes with surface oxygen functionality can make the hybrid electrode surface more accessible to electrolyte ions, acting as conductive paths between porous AC particles. It could be possible to enhance the notable improvement achieved by this study even further by focusing on modifying the surface properties of AC and EEG.
